# Habitat heterogeneity affects the thermal ecology of an endangered lizard

**DOI:** 10.1002/ece3.8170

**Published:** 2021-10-12

**Authors:** Nicole Gaudenti, Emmeleia Nix, Paul Maier, Michael F. Westphal, Emily N. Taylor

**Affiliations:** ^1^ Biological Sciences Department California Polytechnic State University San Luis Obispo California USA; ^2^ Central Coast Field Office US Bureau of Land Management Marina California USA

**Keywords:** activity restriction, burrows, climate change, shade, shrubs, thermoregulation

## Abstract

Global climate change is already contributing to the extirpation of numerous species worldwide, and sensitive species will continue to face challenges associated with rising temperatures throughout this century and beyond. It is especially important to evaluate the thermal ecology of endangered ectotherm species now so that mitigation measures can be taken as early as possible. A recent study of the thermal ecology of the federally endangered Blunt‐nosed Leopard Lizard (*Gambelia sila*) suggested that they face major activity restrictions due to thermal constraints in their desert habitat, but that large shade‐providing shrubs act as thermal buffers to allow them to maintain surface activity without overheating. We replicated this study and also included a population of *G. sila* with no access to large shrubs to facilitate comparison of the thermal ecology of *G. sila* populations in shrubless and shrubbed sites. We found that *G. sila* without access to shrubs spent more time sheltering inside rodent burrows than lizards with access to shrubs, especially during the hot summer months. Lizards from a shrubbed site had higher midday body temperatures and therefore poorer thermoregulatory accuracy than *G. sila* from a shrubless site, suggesting that greater surface activity may represent a thermoregulatory trade‐off for *G. sila*. Lizards at both sites are currently constrained from using open, sunny microhabitats for much of the day during their short active seasons, and our projections suggest that climate change will exacerbate these restrictions and force *G. sila* to use rodent burrows for shelter even more than they do now, especially at sites without access to shrubs. The continued management of shrubs and of burrowing rodents at *G. sila* sites is therefore essential to the survival of this endangered species.

## Graphical abstract

The Blunt‐Nosed Leopard Lizard is a federally endangered ectotherm adapted to California's San Joaquin Desert ecosystem. We compared a population of lizards who had access to shrubs to a population of lizards who had no access to shrubs in the Carrizo Plain National Monument, and found that lizards who do not have access to shrubs thermoregulated more accurately (unexpected), with a trade‐off of being forced underground into cooler burrows for more of the day compared with lizards who had access to shrubs.

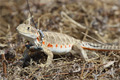

## 1 INTRODUCTION

Many organisms are threatened by the projected increase in global temperatures. As ectotherms, reptiles are disproportionately threatened because their body temperatures are dependent on the temperatures of their environment (Aragón et al., 2010). Models estimate that nearly 40% of lizard populations may be extirpated by 2080 (Sinervo et al., 2010), and heliothermic (sun‐basking) lizards occupying the hottest habitats on the planet could be at particularly high risk because temperatures are already so high. Field observations of microhabitat use paired with comparisons of animals' field‐active and preferred body temperatures to the available microhabitat temperatures can give insight into how an animal uses its thermal landscape (Burrow et al., 2001; Fawcett et al., 2019; Taylor et al., 2021). Such data can also be used to calculate the population's hours of restriction, or the number of hours per day that temperatures in certain microhabitats exceed the animal's preferred body temperature or their upper thermal tolerance and are therefore undesirable or unavailable for use. This information can be used to identify thermal and ecological parameters that may help conserve threatened reptiles and their communities. For example, shrubs and other vegetation are important contributors to the habitat heterogeneity that provides a mosaic of temperatures for effective thermoregulation by lizards (Basson et al., 2017; Goller et al., 2014), suggesting that shrubs may help buffer reptiles from climate change.

**FIGURE 1:**
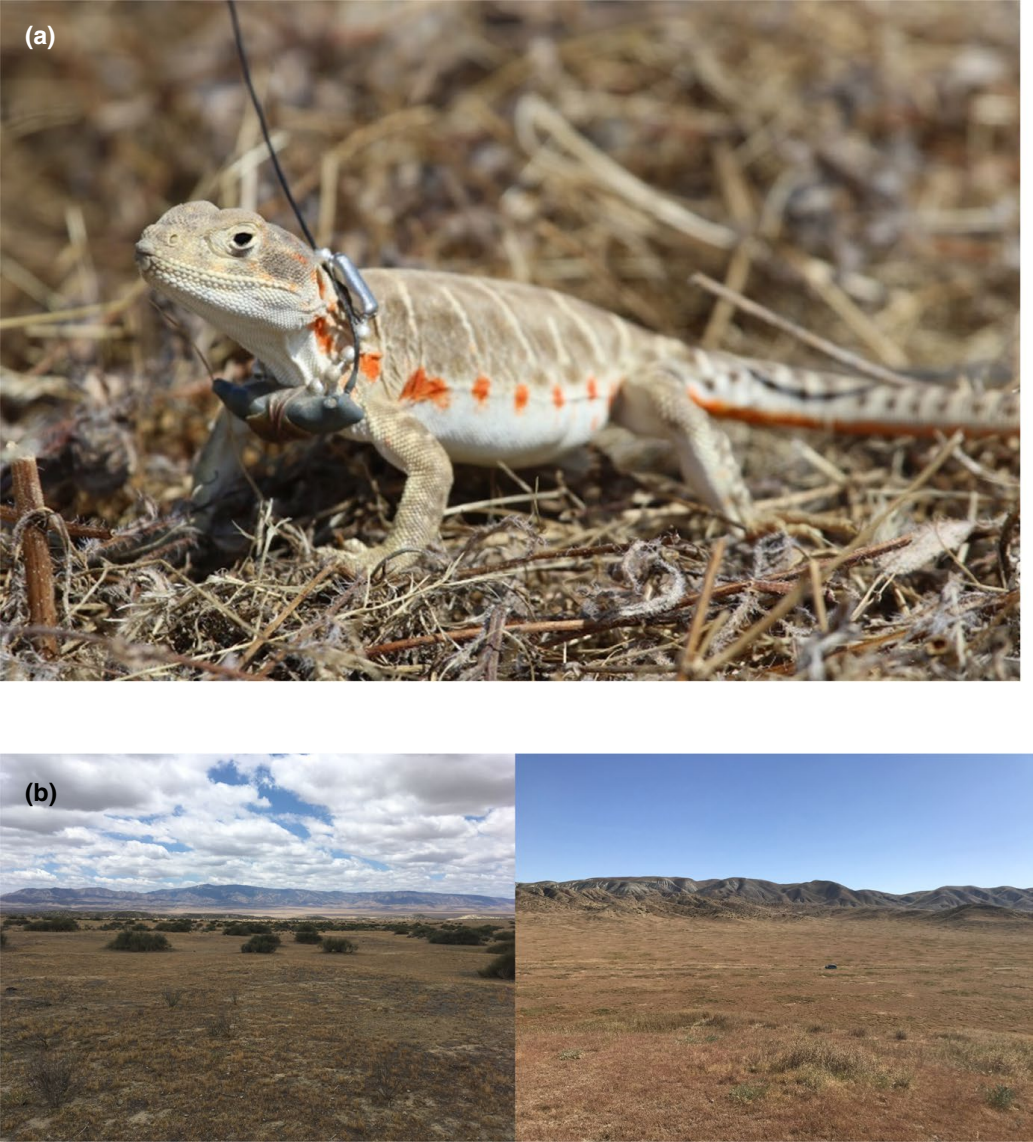
(a) Radio‐collared Blunt‐nosed Leopard Lizard (*Gambelia sila*) in the Carrizo Plain, California, USA. Photo by Emmeleia Nix. (b) The Shrubless (left) and Shrubbed (right) sites on the Elkhorn Plain in the Carrizo Plain National Monument

The Blunt‐nosed Leopard Lizard (*Gambelia sila*) (Figure 1a) is an ectotherm that has been listed as federally endangered since 1967 because almost 90% of the species' historical range has been converted into uninhabitable agricultural fields (U.S. Fish & Wildlife Service, 1998). Once ranging across the vast San Joaquin or California Central Valley, *G. sila* are now restricted to a few small patches of relatively undisturbed San Joaquin Desert habitat. These heliothermic lizards are adapted to the very hot and dry California San Joaquin Desert ecosystem, where already high temperatures are becoming even more extreme (Germano et al., 2011; Ivey et al., 2020). Adult *G. sila* are primarily only active for a quarter of the year (late April through mid‐July) (Germano & Williams, 2005; Montanucci, 1965), during which time they experience high environmental temperatures (Ivey et al., 2020). They feed and breed in this short window, using Giant Kangaroo Rat (*Dipodomys ingens*) burrows for shelter at night and during the heat of the day (Prugh & Brashares, 2012), then entirely retreat into the burrows for most of the remaining nine months of the year. Lizards in many populations, but not all, associate with desert shrubs, including the large gymnosperm shrub *Ephedra californica*. *Ephedra californica* is a foundation species in the San Joaquin Desert community (Lortie et al., 2017) and facilitates the presence of community members, including *G. sila* (Filazzola et al., 2017; Lortie et al., 2017; Westphal et al., 2018), which select for shrubs at fine spatial scales (Germano & Rathbun, 2016).

Until recently, technological constraints have prevented researchers from collecting the continuous body temperature data necessary for studying the thermal ecology of a species such as *G. sila*. Advances in miniaturization and technology of radiotelemetry transmitters now allow for ample data collection on physiological aspects of small animals (Weaver et al., 2021). Ivey et al. (2020) studied the thermal ecology of *G. sila* at a single site with abundant shrubs in 2018 and found that shrubs appear to serve as an important thermal buffer from the heat of the sun, potentially allowing *G. sila* to remain aboveground instead of retreating underground where they would be unable to perform necessary daily activities (Ivey et al., 2020; Westphal et al., 2018). To further test this hypothesis, we studied *G. sila* in 2019 at the same site as Ivey et al. (2020), hereafter called Shrubbed, and added a second nearby site where *G. sila* had virtually no access to shrubs (Shrubless). This allowed us to further assess the importance of shrubs for thermoregulating *G. sila* that were experiencing otherwise similar environmental conditions, and therefore understand how important shrubs may be in ensuring this endangered species' survival. If shrubs provide a thermoregulatory benefit to *G. sila*, then lizards with access to shrubs should be active aboveground longer and use rodent burrows less often during the day than lizards without access to shrubs. Additionally, lizards with access to shrubs should thermoregulate more accurately (i.e., field‐active body temperatures closer to preferred body temperatures; Hertz et al., 1993) than lizards without access to shrubs, and should have fewer hours of restriction currently and in modeled future scenarios when ambient temperatures will rise. Identifying aspects of the environment, such as shrubs, that may help *G. sila* thermoregulate more efficiently is important for informing management efforts to protect this species and other sensitive San Joaquin Desert species from rising temperatures in some of the hottest, driest parts of the continent.

## 2 MATERIALS AND METHODS

### 2.1 Field sites and study species

A pair of sites, one dominated by *E. californica* and other smaller perennial shrubs (hereafter named Shrubbed), and the other with no *E. californica* and very few other shrubs (Shrubless), were selected on the Elkhorn Plain within the Carrizo Plain National Monument in California, USA (Figure 1b). Shrubless was selected because lizards had been seen in the area previously, and it was only 6.5 km away from Shrubbed where we have previously collected data. The sites are similar in size (400 m^2^), as well as climate and elevation. Microhabitat use and shrub association of lizards at Shrubbed were studied in 2016 (Westphal et al., 2018), and field‐active body temperatures of lizards were regularly recorded there in 2018 (Ivey et al., 2020). *Gambelia sila* at Shrubbed had access to ample shade provided primarily by the aforementioned large *E. californica* shrubs. Shade was also available from smaller perennials such as *Isocoma acradenia* and *Gutierrezia californica* and small annual plants such as *Amsinckia* sp. and nonnative grasses. In contrast, lizards at Shrubless had limited access to aboveground shade, which was provided by very few *I. acradenia*, *G*. *californica,* and *Astragalus* sp. (mostly *A. lentiginosus*, sometimes *A. oxyphysus*), in addition to small annual forbs and grasses. Shrubless had only a few individual perennial shrubs in the entire site, and notably, these shrubs were only used by a total of two lizards whose territories happened to overlap with these shrubs. Therefore, the use of shrub‐provided shade by lizards at Shrubless was extremely rare (see Results). Lizards at both sites had access to burrows, which were confirmed to be engineered by *D. ingens* from 5 nights of trapping with 61 traps at each site in August 2020. *Dipodomys ingens* were captured at both sites, with very small numbers of *D. nitratoides* at Shrubbed exclusively.

We captured twenty lizards at each site (*N* = 40 total) by handheld lasso over the course of three days in late April 2019, and collected the following data for each lizard: sex, reproductive state in females (gravid or not), snout–vent length (SVL, ±0.5 mm), and mass (±1 g). Lizards were fitted with VHF temperature‐sensitive radiotransmitter collars with 16 cm whip antenna (Holohil Model BD‐2T Holohil Systems Ltd, Carp, Ontario, Canada, attached with epoxy to ball chain “collars”) following the methods of Ivey et al. (2020), then released at their site of capture the same day. Throughout the season, several lizards lost their collars, and these collars were placed onto new lizards, such that a total of 47 individual lizards (*N* = 22 Shrubbed, *N* = 25 Shrubless) were tracked from May through mid‐July 2019 for an average of 53 ± 12 days. Those that lost collars likely represented predation events, although in some cases, collars could have slipped off. In addition to the lost collars that were recovered, four lizards and their collars disappeared (likely from being carried away by avian predators) or were lost deep in burrows (where the collar was excavated at the end of the season). Lizards with less than two weeks of valid temperature data were excluded from analyses. The final dataset included the following sample sizes: May—Shrubbed: *N* = 16, Shrubless: *N* = 17; June—Shrubbed: *N* = 16, Shrubless: *N* = 18; and July—Shrubbed: *N* = 16, Shrubless: *N* = 15.

### 2.2 Microhabitat use

We tracked *G. sila* using a VHF receiver (R‐1000 Telemetry Receiver, Communications Specialists, Inc., Orange, CA, USA) and 3‐element Yagi antenna. Each lizard was tracked 1–2 times per day for six days per week over the course of their active season, from May through mid‐July. The observations at both sites were evenly distributed among morning, midday, and afternoon, and the lizards were tracked in a random order to ensure that observations were spread out throughout the day. Each lizard's microhabitat use was recorded as one of the following: in the shade of a plant (with plant species identified), in full sunlight, or underground in a burrow. A lizard was designated as underground in a burrow if they were not visible from the burrow mouth; sometimes, lizards sat close to the entrances of burrows, but this was categorized as the open because most of their body, notably the temperature‐sensitive radio collar, was in sunlight. We then calculated the percent of time *G. sila* used each microhabitat in May, June, and July at each site. To compare the probability that a lizard would be found underground (in burrows) between the two sites, we ran a mixed‐effects logistic regression model in R (R Core Team, 2020; RStudio, 2020, lme4 package v. 1.1‐26, Bates et al., 2015) with time as a polynomial, site and month as fixed effects, and lizard ID as a random effect.

At the end of the active season, we collected data on *D. ingens* burrow densities at each site by counting the number of active or recently inactive burrows (Bean et al., 2012) within 10 m along four 100‐m randomly placed transects at each site. We compared the burrow densities at the Shrubbed and Shrubless sites with Welch's *t*‐test in R. We also collected data on perennial shrub densities by counting the number of perennial shrubs in a 10‐m radius around 16 random points (Zuliani et al., 2021) at each site, and compared the densities with Welch's *t*‐test in R.

### 2.3 Temperature variables

At the center of each site, we installed a stationary 3‐m tall solar‐powered (Tycon RemotePro 2.5 W Solar Power System with Vikram Solar Eldora 10P solar panel) omni‐antenna (Telonics Model RA‐6B) and receiver with data acquisition system (Telonics TR‐5 Option 320). We estimated the range for continuous, gap‐free data collection with this antenna to be approximately 300 m. About every five minutes, the receiver logged the interpulse interval of the signal from each radio collar in range, and we downloaded these data from the receivers each week. Because the radiotransmitters were externally attached to the lizards, *T*_b_ values may represent an overestimation of core *T*_b_ because they can heat rapidly from solar radiation; however, surgical implantation of radiotransmitters is not possible in an endangered species such as *G. sila*. We used manufacturer‐provided calibration curves and the program Vinny Graphics v2.07 to convert the interpulse intervals to field‐active body surface temperatures, which act as estimates of lizard body temperature (*T*_b_). Prior to analysis, we removed any outliers greater than two standard deviations away from each lizard's mean *T*_b_, as these likely represented glitches in the data acquisition system; such outliers were uncommon (<5% of data points).

To collect data on the environmental temperatures of the three available microhabitats to these lizards for the entirety of the study, we deployed lizard physical models in sunlight, in the shade, and inside burrows, using the same models as Ivey et al. (2020). Models consisted of copper pipes (2.5 cm diameter and 12 cm long) capped with PVC and spray‐painted matte gray and matte tan to resemble the color of the lizards' skin. Models that were placed under shrubs and in the open were given two “legs” in the form of metal wire looped around the pipes so they could be propped up to resemble *G. sila* resting posture. Each model housed a Thermochron iButton (DS1921G‐F5) programmed to record temperature every hour, on the hour. While empty models provide instantaneous operative temperature, we chose to fill the models with water to mimic a body cavity (Dzialowski, 2005) and to replicate the exact methods of Ivey et al. (2020); we also added plumber's tape before screwing on the caps to maintain watertight seals. We placed the models haphazardly at each site (Shrubbed: *N* = 4 under *E. californica* shrubs, *N* = 4 in the open, and *N* = 4 anchored about 0.5 m inside the mouths of burrows; Shrubless: *N* = 4 in the open and *N* = 4 anchored inside the mouths of burrows). Models inside *D. ingens* burrows and under shrubs received little to no solar radiation, whereas models in the open were exposed to full sunlight during daylight hours. The models under shrubs and in the open were placed facing north, south, east, and west, and the orientations of the burrow mouths were recorded. Every two weeks, we downloaded the iButton data using OneWireViewer (Maxim Integrated), refilled the models with water, and returned them to the same locations. Physical model temperatures in the three microhabitats were treated as operative temperatures (*T*_e_) in analyses (see below), where *T*_e_ represents the effective microhabitat temperatures available to *G. sila*.

### 2.4 Preferred body temperature and thermoregulatory accuracy

As *G. sila* aestivation approached in mid‐July, we recaptured and reprocessed each lizard and removed their collars. Before returning each lizard to its capture site, we collected data on its preferred body temperature (*T*_set_) in a thermal gradient as described in Ivey et al. (2020). The gradient consisted of 3 lanes (250 × 20 × 25 cm) filled with sand substrate and separated by wood dividers, ranging from 47°C at the hot end to 10°C at the cool end. Three *G. sila* were placed into the center of the gradient at a time, each in its own lane, with thermocouples (Model 5SRTC‐TT‐K‐40‐72; Omega Engineering, UK) in their cloacae recording body temperature every 10 min for three hours. These data were recorded on a data logger (Model RDXL4SD; Omega Engineering, Egham, Surrey, UK), and only the last hour of data was used for analysis.

We calculated average *T*_set_ for each of the two populations after removing outliers greater than 2 standard deviations away from each lizard's mean, and we used the interquartile range (IQR) of each population as its *T*_set_ range. Since there was no significant difference in *T*_set_ between the two populations (see Results), we used the mean *T*_set_ IQR of all lizards for the following analyses. We calculated lizard thermoregulatory accuracy (*d*_b_) by subtracting the mean *T*_set_ IQR from each instance of *T*_b_ in the field (Hertz et al., 1993). When *T*_b_ fell within *T*_set_ IQR, *d*_b_ was zero. Either very high positive or very low negative values of *d*_b_ represented poor thermoregulatory accuracy because the field‐active *T*_b_ was higher or lower than *T*_set_ range. Lizard *T*_b_ was also compared with the panting threshold (*T*_pant_) of *G. sila*, a measure of upper thermal tolerance that Ivey et al. (2020) measured in 2018. All *d*_b_ values for each lizard were averaged by hour per day from 0,700 to 2,000 (daylight hours when lizards can actively thermoregulate), then each hour's *d*_b_ values were averaged to create hourly *d*_b_ values per month. To compare the thermoregulatory accuracy of *G. sila* at Shrubbed and Shrubless, *d*_b_ values were further averaged to give one value per lizard per month. We then performed a multifactor ANOVA with *d*_b_ as the response variable; site, month, and the site x month interaction as fixed factors; and lizard ID as a random factor nested within site, using JMP (SAS Institute Inc., v. 14.3, 2018).

### 2.5 Hours of restriction and climatic projections

We compared temperatures from the physical models (*T*_e_) to *G. sila T*_set_ and *T*_pant_ each hour of the day for each month to calculate the number of hours in a day that a given microhabitat would be thermally stressful (i.e., exceed either *T*_set_ or *T*_pant_) for a lizard. We designated hours of restriction as “basking restriction” when temperatures in open sunlight were too hot and lizards therefore must remain in shade or in burrows; “aboveground restriction” when temperatures in the open and shade of large shrubs were too hot and lizards therefore must retreat to burrows (this is only applicable for lizards at Shrubbed); and “total restriction” when all three microhabitats including burrows were too hot (Ivey et al., 2020).

Each of these hours of restriction variables was then recalculated by adding 1°C and 2°C to the *T*_e_ values for each microhabitat, following the methods of Ivey et al. (2020) which used the Cal‐Adapt representative concentration pathway (RCP) climate scenarios 4.5 and 8.5 to determine that 1‐2°C represent likely mean temperature increases this century in the Elkhorn Plain (California Energy Commission, 2019).

## 3 RESULTS

### 3.1 Microhabitat use

**FIGURE 2:**
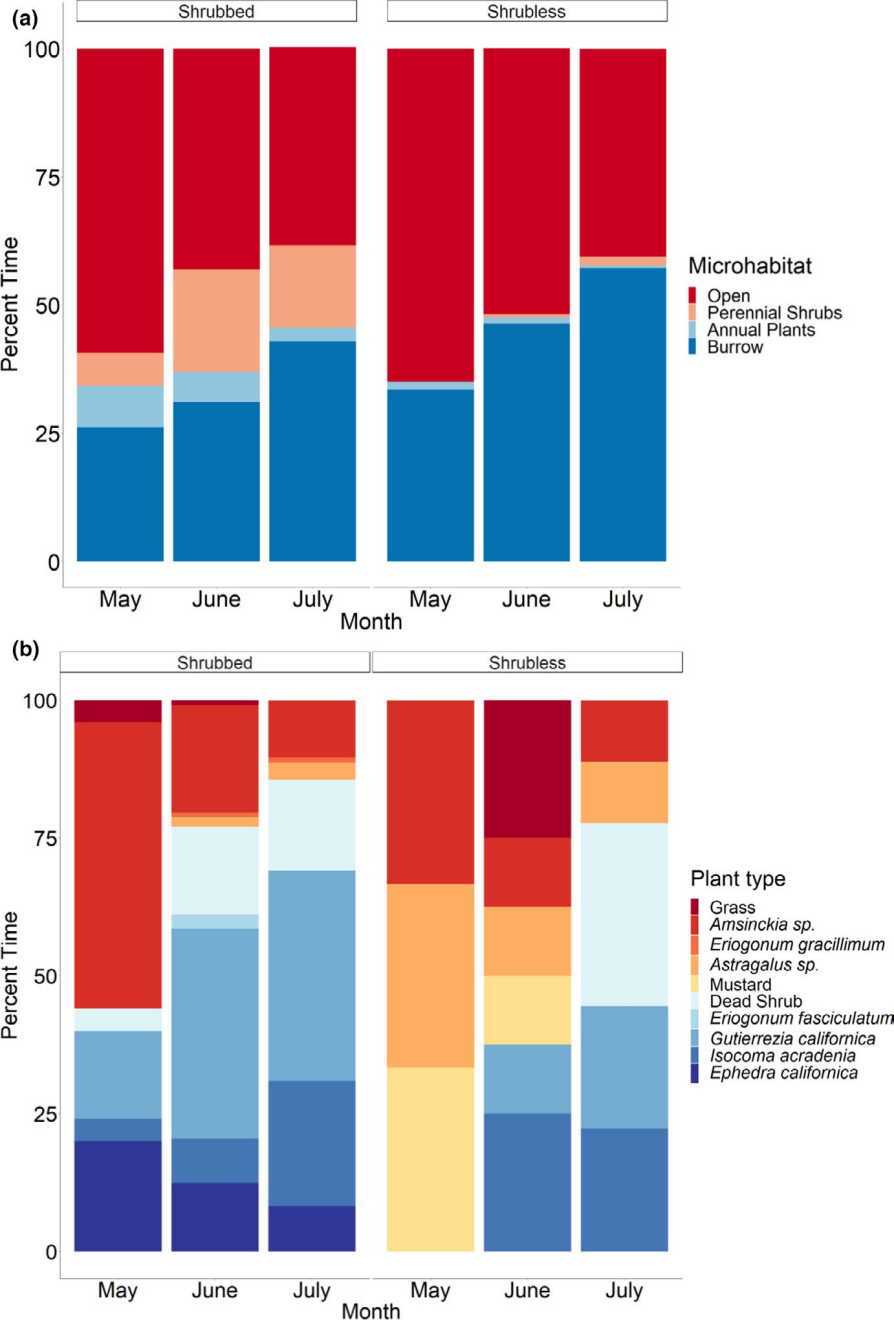
Microhabitat use of *Gambelia sila* at Shrubbed and Shrubless over the course of their 3‐month primary active season in 2019, showing (a) percent of time spent in the open sun, in the shade of annual and perennial plants, and in rodent burrows, and (b) use of plant species for shade at each site. Lizards at Shrubless spent more time inside burrows and less time in the shade of plants, and the plants they used were often annuals because woody shrubs were extremely rare at that site

From May through mid‐July 2019, we collected 1,148 individual radiotelemetry observations of *G. sila* at Shrubbed and 1,019 observations of lizards at Shrubless. Shrub density was significantly different between the two sites (Shrubbed: 15.69 ± 4.02 shrubs/987 m^2^, Shrubless: 0.56 ± 0.22 shrubs/987 m^2^; *t* = 3.76, *p* = .002). In May, lizards at both sites spent the majority of daytime hours basking in the open (Figure 2a). In June and July, lizards at both sites spent progressively less time in the open and more time in the shade of plants and in burrows than they did in May. Although some lizards at Shrubless found some shade from sparse annual plants and shrubs, they collectively spent very little time in the shade throughout the active season because shade was largely unavailable. In June and July, lizards from Shrubless spent 46% and 57% of their observed time, respectively, inside burrows, compared with 31% and 43% for lizards at Shrubbed. The probability that lizards at Shrubless would be found underground in *D. ingens* burrows instead of aboveground was higher than that for lizards at Shrubbed (*z* = 4.35, *p* < .001) throughout the season. Burrow density was not significantly different between the two sites (Shrubbed: 35.83 ± 4.71 burrows/100 m, Shrubless: 44.67 ± 6.26 burrows/100 m; *t* = −1.36, *p* = .23). Lizards at both sites most likely spent all their time in burrows at night.

The woody perennial shrubs most commonly used for shade by lizards at Shrubbed were *G*. *californica*, followed by *I*. *acradenia*, *E. californica,* and unidentifiable dead small shrubs, which were likely either *I. acradenia* or *G*. *californica*. One individual had access to and used *E*. *fasciculatum* (Figure 2b). In May, when annuals were plentiful, lizards at Shrubbed used the shade of *Amsinckia* sp. 52% of the time they were in shade, and this decreased to 19% and 10% in June and July, respectively, when lizards started using woody shrubs more often for shade (Figure 2b). Lizards at both sites also used annual or perennial *Astragalus* sp., as well as the annual forb *E*. *gracillimum* and nonnative grasses (primarily *Schismus* sp. and *Bromus* sp.) for shade.

### 3.2 Thermoregulation

The mean *T*_set_ for *G. sila* at Shrubbed was 34.1°C with IQR of 32.3–36.8°C, and mean *T*_set_ for lizards at Shrubless was 35.0°C with IQR of 35.1–38.5°C. Because these values were not significantly different from one another (*t* = −0.89, *p* = .38), they were pooled to create a single *T*_set_ IQR of 33.2–37.9°C for the *G. sila* in this study. This IQR is very similar to the IQR of 32.3–37.5°C used by Ivey et al. (2020).

**FIGURE 3:**
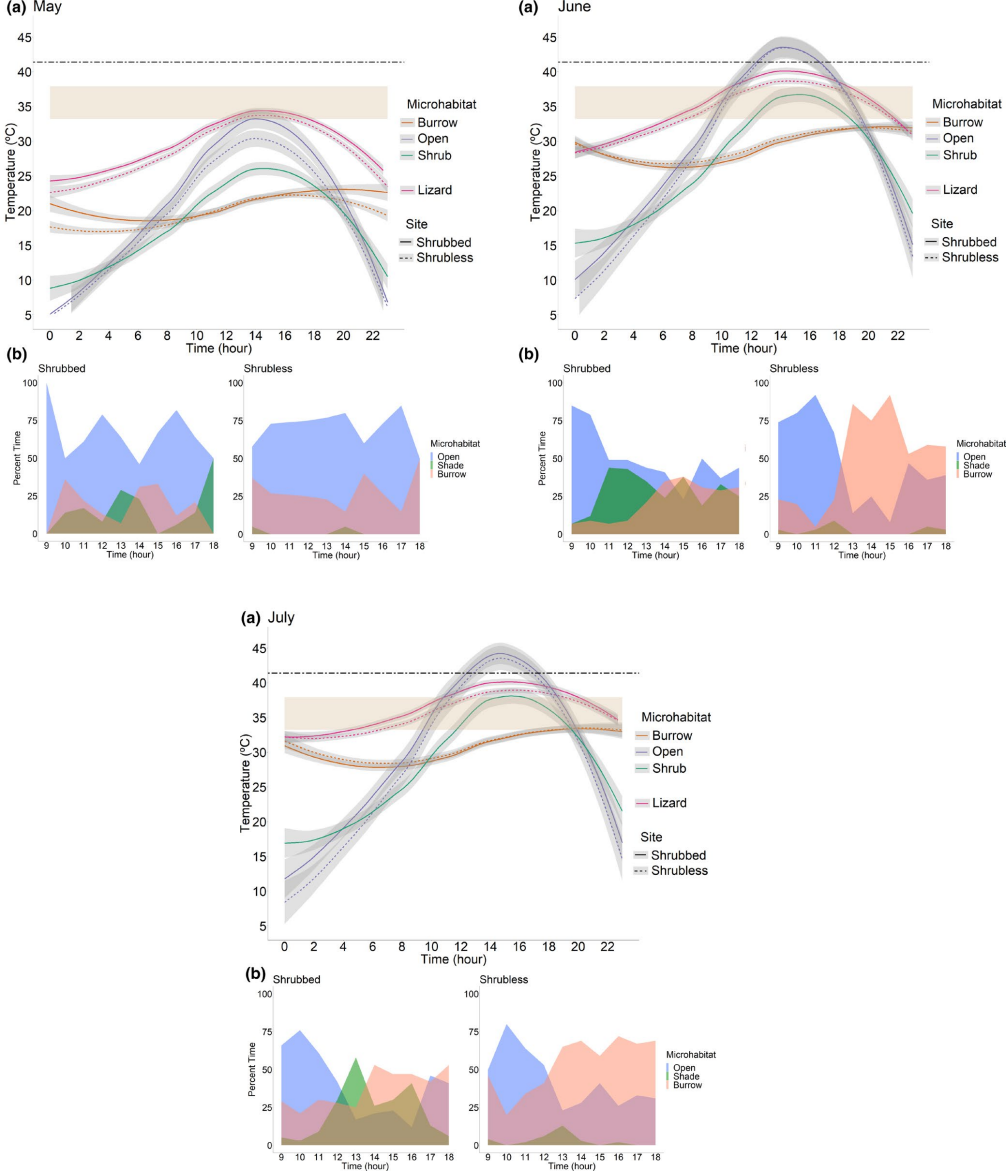
(a) Field‐active body temperatures of *Gambelia sila* at a Shrubbed site and a Shrubless site in May, June, and July 2019, with gray shading representing 1 *SEM*; operative temperatures of three microhabitats (open sunlight, shade of plants, and rodent burrows); the dark lines represent means, the tan bar represents the lizard *T*_set_ range as measured in a thermal gradient; and the dotted line is the panting threshold of *G. sila* (from Ivey et al., 2020). (b) The percent of observations in which lizards used each of the three microhabitats at each site for each month during daylight hours

**FIGURE 4:**
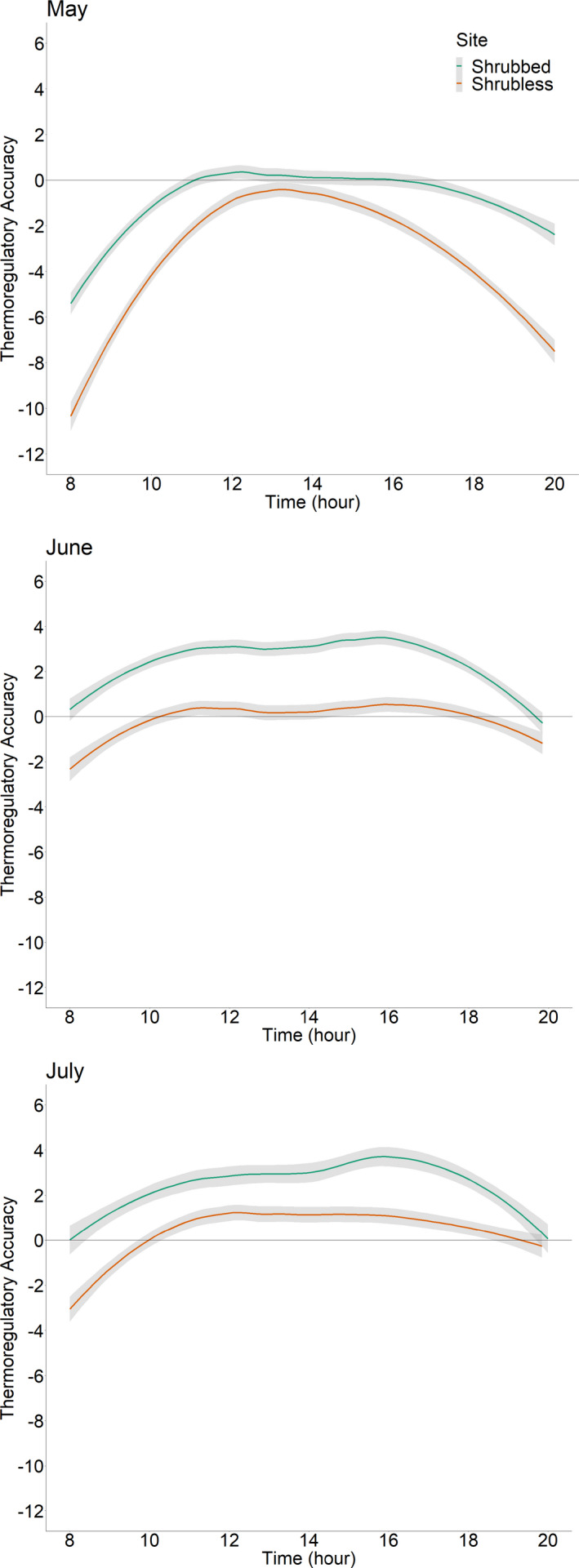
Thermoregulatory accuracy (*d*_b_) of *Gambelia sila* at a Shrubbed site (orange) and a Shrubless site (blue) during daylight hours over the course of their 3‐month primary active season in 2019, with gray shading representing 1 *SEM*. The line at zero represents lizards thermoregulating within *T*_set_; positive values mean that lizards are thermoregulating above the upper bound of their *T*_set_ range; negative values mean that lizards are thermoregulating below the lower bound of their *T*_set_ range. During the hottest months of June and July, lizards from Shrubbed had poorer thermoregulatory accuracy than lizards from Shrubless

*Gambelia sila* maintained *T*_b_ within their *T*_set_ during daylight hours in the month of May, but in June and July, their mean *T*_b_ slightly exceeded *T*_set_ for a majority of their active daytime hours (Figure 3), resulting in good *d*_b_ in May and poorer *d*_b_ in the hotter months of June and July (Figure 4). The mean *T*_b_ of *G. sila* at each site never exceeded *T*_pant_, although in June and July, the *T*_e_ in open sunlight exceeded *T*_pant_ for several hours, while the shrub and burrow *T*_e_ stayed below *T*_pant_ (Figure 3). *T*_b_ of lizards at Shrubless was slightly lower than at Shrubbed but not significantly so (site: *F* = 2.74, *df* = 1, *p* = .10; month: *F* = 243.15, *df* = 2, *p* < .0001; site‐by‐month interaction: *F* = 0.27, *df* = 2, *p* = .76; Figure 3). As a day progressed, lizards moved from burrows to the open and then retreated under vegetation or back into burrows typically in the late afternoon when temperatures were highest (Figure 3). On average, lizards at Shrubless thermoregulated more accurately than lizards at Shrubbed (site: *F* = 77.39, *df* = 1, *p* < .0001; month: *F* = 193.71, *df* = 2, *p* < .0001; site‐by‐month interaction: *F* = 0.12, *df* = 2, *p* = .89; Figure 4). In May, lizards at both sites thermoregulated fairly accurately (*d*_b_ near 0 in the middle of the day), but lizards at Shrubbed thermoregulated more accurately than lizards at Shrubless (Figure 4). In June and July, *G. sila* at Shrubless thermoregulated more accurately than lizards at Shrubbed. During these hot months, *d*_b_ of lizards at Shrubbed was better in the mornings and evenings but poorer during the day, whereas lizards at Shrubless kept their *d*_b_ closer to 0 during the day by staying in burrows more often than lizards at Shrubbed.

### 3.3 Hours of restriction and climatic projections

**FIGURE 5:**
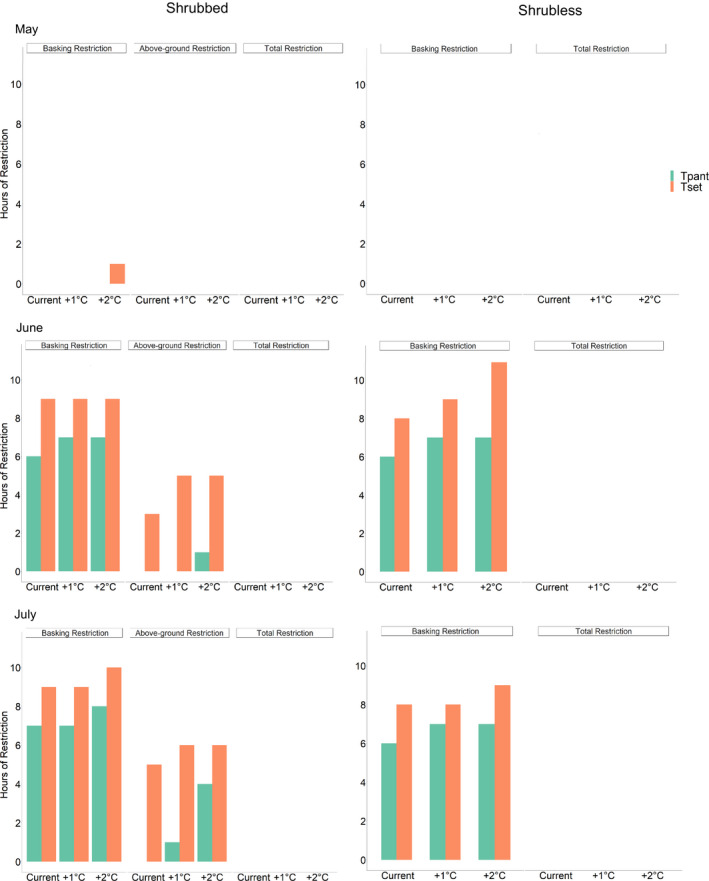
Hours of restriction from using specific microhabitats for *Gambelia sila* at a Shrubbed Site and a Shrubless Site over the course of their 3‐month primary active season in 2019, calculated as the number of daylight hours in which microhabitat operative temperature *T*_e_ exceeds *T*_set_ (orange) or *T*_pant_ (green). Current data show estimates from 2019, and +1°C and +2°C data model increases in temperature due to climate change. In general, lizards at Shrubbed experienced about one more hour of restriction than lizards at Shrubless

Because May temperatures are so mild, *G. sila* do not currently experience any hours of restriction from using various microhabitats during daylight hours in May (Figure 5). However, in June and July, *G. sila* are restricted from basking in sunlight (basking restriction) for 8–11 daylight hours because *T*_b_ exceeds *T*_set_, or for 6–8 daylight hours because *T*_b_ exceeds *T*_pant_ (Figure 5). In June and July, *G. sila* at Shrubbed experience one more hour of basking restriction than lizards at Shrubless (Figure 5). Lizards at Shrubbed are completely restricted from being aboveground (aboveground restriction) for 3 of the 12 hr in June and for 8 hr in July because *T*_b_ exceeds *T*_set_. Currently, *T*_e_ inside burrows at both sites never exceeds *T*_set_ or *T*_pant_, and *T*_e_ under shrubs at Shrubbed never exceeds *T*_pant_.

As expected, adding 1°C and 2°C to the *T*_e_ data resulted in additional projected hours of restriction associated with climate change for both populations in June and July (Figure 5). Lizards at Shrubbed will be restricted from basking in sunlight and thus staying within their *T*_set_ range for 9–10 daylight hours, and lizards at Shrubless will be similarly restricted for 8–11 hr. Notably, lizards at Shrubbed should still be able to stay aboveground for several hours under future climate change scenarios because of their access to the shade of a shrub, while lizards at Shrubless do not have this option. Even under conditions positing 1°C or 2°C increases in all microhabitats, temperatures in burrows should remain low enough for lizards to stay below their *T*_set_.

## 4 DISCUSSION

As predicted, we found that *G. sila* that had access to shrubs spent more time aboveground than those that did not, as lizards at Shrubless spent more time inside *D. ingens* burrows. However, unexpectedly, the presence of shrubs did not give *G. sila* higher thermoregulatory accuracy. This was mainly because staying inside burrows for longer periods of time actually allowed lizards to remain closer to their preferred body temperature, suggesting a trade‐off between thermoregulation and activity aboveground. There was no difference in *D. ingens* burrow density between the two sites, indicating that the higher frequency of burrow use by *G. sila* at Shrubless was not the result of more available burrows. Instead, lizards at Shrubless likely have to limit their time spent aboveground because they would become too hot in the open sunlight, while lizards at Shrubbed can retreat to the shade of a shrub when the open microhabitat becomes too hot.

Like Ivey et al. (2020), we found that *G. sila* will be further constrained from being active aboveground under future climate change scenarios, with temperatures undesirable (above preferred) or unlivable (above thermal maximum) for many hours per day. This constraint, however, is mitigated by shrubs, as lizards with access to shrubs could remain aboveground for several hours longer than lizards with no such access. Taken together, our study shows that shrubs are important in buffering *G. sila* from the effects of high temperatures, but *D. ingens* burrows remain the most essential refugia from high temperatures both now and in the future.

### 4.1 Microhabitat use—activity aboveground

The presence of shrubs allowed *G. sila* to spend more time aboveground, potentially enabling them to continue patrolling for mates, looking for prey, or engaging in other activities. Although it is unknown whether *G. sila* can hunt and/or mate underground, typically heliothermic, diurnal lizards conduct the majority of these behaviors aboveground. A critically endangered lizard in Australia, the Pygmy blue‐tongue lizard (*Tiliqua adelaidensis*) spends the majority of its time underground inside burrows but still needs to exit its burrow to feed (Milne et al., 2003). This lizard has likely evaded extinction thus far due to the tolerable temperatures inside burrows, and artificially added burrows have increased their density (Souter et al., 2004). Burrows constitute crucial thermal refugia for other lizard species inhabiting hot, arid regions worldwide, and their importance is even more critical as temperatures rise (Fenner et al., 2012; Grillet et al., 2010; Moore et al., 2018). Models suggest that lizards will need to go deep into burrows to deal with climate change (Kearney & Porter, 2020). However, aboveground shade may also be critical to facilitate feeding, mating, and other behaviors in species such as *G. sila*. Crotaphytid lizards hunt their prey using visual cues and lack lingually mediated prey chemical discrimination (Cooper et al., 1996), suggesting that most hunting indeed occurs aboveground. Male crotaphytids rely on bright mating coloration to find mates (Baird, 2004), with chemosensory cues from femoral secretions appearing to play secondary roles such as permitting female assessment of male quality (Baird et al., 2015). Shrubs may therefore play a critical role in allowing *G. sila* to hunt, find, and court mates, and defend territories, especially as temperatures in the open continue to rise. In our study, we did not examine whether there were consequences for the lizards spending less time aboveground at Shrubless in terms of hunting success or fitness. Such a study would further elucidate the importance of shrubs in allowing aboveground activity in *G. sila*.

### 4.2 Microhabitat use—shade

As the season progressed and the temperatures rose, the importance of shade increased for *G. sila* at both sites (Figure 2a). Lizards mostly used annuals early in the season when annual cover was thick and then used perennials more often as time went on (Figure 2b). Dense grasses reduce locomotion speed in lizards (Newbold, 2005), and *G. sila* prefer open ground (Warrick et al., 1998) and tend to avoid areas with invasive annual grasses (Filazzola et al., 2017; Germano et al., 2001; Hacking et al., 2013). However, our study shows that when shrubs are not available, *G. sila* can use annuals for shade. We did not place models underneath annuals to assess the thermal quality of this microhabitat, an excellent topic for future study. Although many ectotherms avoid areas with invasive grasses, the microhabitats under these grasses may actually be cooler than an undisturbed area and may theoretically provide a better thermal environment as temperatures rise (Garcia & Clusella‐Trullas, 2019). In addition, we observed *G. sila* climbing annuals including grasses, especially at Shrubless, which could be a way to escape high surface temperatures or to gain a better view of the surroundings. *Astragalus* sp. were used much more often as shade by lizards at Shrubless than those at Shrubbed (Figure 2b) even though there were abundant *Astragalus* sp. at both sites. This may be because *Astragalus* sp. was the most abundant plant available for shade for lizards at Shrubless, which otherwise had only very sparse *I. acradenia* and *G*. *californica* and no *E. californica*. Surprisingly, *E. californica* was not the predominant shrub used by *G. sila* at Shrubbed in our study, which used *I. acradenia* and *G*. *californica* more often.

Numerous studies at Shrubbed in previous years documented more extensive use of *E. californica* by *G. sila* (Ivey et al., 2020; Lortie et al., 2020; Westphal et al., 2018). Westphal et al. (2018) showed that *G. sila* select for large shrubs such as *E. californica* more than what would be expected based on shrub density, and Filazzola et al. (2017) showed that *G. sila* scat is found more frequently under *E. californica* canopies than in the open. Our study followed a relatively wet winter, and the smaller *I. acradenia* and *G*. *californica* shrubs may not have been as present during the studies conducted in previous years. The understories of *E. californica* were also smothered with tall nonnative grasses capitalizing on the shade provided by the shrub, which likely prevented *G. sila* from using them for shade as often as in previous years (Filazzola et al., 2017; Ivey et al., 2020; Westphal et al., 2018). This observation suggests that *G. sila* are flexible and can use shade from any plant, not just *E. californica*, which is important information for habitat management and restoration efforts.

Qualitatively, from our telemetry observations, the *G. sila* at Shrubbed seemed to use smaller perennial shrubs such as *I. acradenia* and *G*. *californica* more often than *E. californica* for thermoregulatory purposes, and instead seemed more likely to retreat to *E. californica* if they felt threatened. Large, dense shrubs provide lower temperatures than small shrubs (Kerr et al., 2008), but *G. sila* appear to use burrows when temperatures become really high. *Gambelia sila* may prefer to use smaller shrubs, when available, for thermoregulatory purposes because they provide cover from solar radiation with less obstruction of surrounding views, allowing these visually oriented lizards to better see prey, predators, mates, and rivals.

### 4.3 Thermoregulation

Thermoregulatory accuracy was higher for lizards at Shrubless than at Shrubbed, which was unexpected because we predicted that the ability to utilize shrubs would improve the thermoregulatory accuracy of *G. sila*. However, our result is consistent with the observation that *T*_e_ in the open was higher at Shrubbed than at Shrubless (Figure 3), even though we chose these nearby sites as “matched” sites. Models inside burrows also warmed up faster in the morning at Shrubbed than at Shrubless in May, but not in June or July (Figure 3). The temperature variation between sites may reflect soil composition, reflectance, or other variables (Limb et al., 2008). Our results suggest that very small differences in environmental temperatures can impact body temperature and thermoregulatory accuracy in heliothermic lizards, and emphasize the importance of understanding the thermal landscape of a given environment (Milling et al., 2018), which has been shown via models to impact thermoregulation (Sears et al., 2016).

Another contribution to the better thermoregulatory accuracy of *G. sila* at Shrubless is that they spent more time in burrows during the middle of the day (Figure 2), where *T*_e_ is closer to *T*_set_, while lizards at Shrubbed spent more time aboveground, both in open sunlight and in the extensive shade that is unavailable at Shrubless. It is possible that lizards at Shrubbed were able to risk operating at *T*_b_ higher than their *T*_set_ during the day because they have an available aboveground buffer in the form of ample shade, while lizards at Shrubless have to limit their time spent aboveground because they cannot risk becoming too hot before retreating into a burrow. Simulated models indicate that lizards are expected to conserve energy by thermoconforming in more homogeneous landscapes such as Shrubless (Basson et al., 2017); the lizards at Shrubless indeed spent less time in sunlight and therefore were more thermoconforming than lizards at Shrubbed. Notably, our *T*_set_ values may underestimate the true *T*_set_ of *G. sila*, given that we could only measure *T*_set_ for three hours and could not afford time to allow lizards extensive acclimation inside the gradient.

### 4.4 Predation risk and other site differences

The lack of shrubs at Shrubless may have consequences that extend beyond thermoregulation. More *G. sila* at Shrubless (*N* = 6) were lost to probable predation than at Shrubbed (*N* = 1). Indeed, there were more confirmed mortalities (dead lizard found with collar) at Shrubless (*N* = 4) than at Shrubbed (*N* = 1); some of these lizards had missing limbs, but otherwise, their bodies were mostly intact. Lost collars were likely lizards that were carried away by birds, which are common predators of *G. sila* (Germano, 2019). In addition, two collars at Shrubless were found with lizard entrails, suggesting that those lizards were killed by avian predators (Germano, 2019; Nelson, 1934). While sample sizes of dead and lost *G. sila* are too small to draw definitive conclusions, these data suggest that lizards at Shrubless might experience higher predation pressure than those at Shrubbed. Lack of large shrubs such as *E. californica* may allow birds of prey or other visually oriented predators such as snakes to more easily see and capture lizards on the desert floor. Predation may therefore be an additional reason why *G. sila* at Shrubless spent more time underground in rodent burrows than those at Shrubbed. Predator avoidance was found to be an even higher priority for lizards in choosing a microhabitat than thermoregulation in Velvet geckos (*Oedura lesueurii*, Downes & Shine, 1998), and Mediterranean lizards (*Psammodromus algirus*) avoided leafless shrubs in early spring because they could not hide from predators as easily (Martín & López, 1998). In accordance with this idea, *G. sila* were observed using *E. californica* for predator avoidance in our study and in others (Filazzola et al., 2017; Montanucci, 1965; Westphal et al., 2018).

There are many other factors that may contribute to differences in activity and thermoregulation between lizards at our two sites, which we did not explicitly measure for this study. Abundance and composition of small arthropods that serve as the lizards' prey (Germano et al., 2007) may be different between the two sites, especially since the vegetation composition is so different. There is also the possibility that soil composition is different between the two sites; anecdotally, the soil at Shrubbed is rockier than at Shrubless. This may have contributed to thermal differences on the ground that impacted lizard thermoregulation.

### 4.5 Hours of restriction and climatic projections

Our analysis of hours of restriction confirms the conclusion of Ivey et al. (2020) that *G. sila* are already thermally stressed, in that high temperatures force them to spend many hours in shade or inside burrows. Hours of restriction based on *T*_pant_ were about 1 hr fewer on average compared with Ivey et al. (2020), and this is likely because their 2018 field season, and therefore their *T*_e_ used for analysis, occurred during a warmer summer than in 2019. With the anticipated increases of 1 or 2°C due to climate change, *G. sila* will likely face additional restriction during their active season. While lizards at both sites have relatively similar projected hours of restriction, lizards at Shrubbed will have on average one more hour of basking restriction than lizards at Shrubless. These data present an interesting conundrum: *G. sila* at Shrubless do not have aboveground shelter from the sun and from predators and therefore must spend more time inside burrows, but the slightly cooler temperatures on the open desert floor at Shrubless suggest that lizards there may actually experience fewer hours of restriction from basking in sunlight than lizards at the hotter Shrubbed site. However, lizards at Shrubbed still have the option of staying aboveground for more hours of the day than lizards at Shrubless because they can retreat to the cooler shade of shrubs.

Further increases in the number of hours of basking restriction or aboveground restriction are problematic because these lizards already are only active for about three months a year. Their ability to compensate for climate change by becoming active earlier in the year is limited because their activity would be stymied by the dense invasive annual vegetation that appears in February–March and only begins to be clipped by *D. ingens* and/or grazed by cattle in May as these lizards emerge from aestivation. Fortunately, our data suggest that *G. sila* are unlikely to be restricted from all their microhabitats even after a 2°C increase. Also, our projections merely added 1–2°C to current *T*_e_, whereas certain microhabitats might actually warm at a slower rate, providing thermal buffers (Baust, 1976; González‐del‐Pliego et al., 2020; Scheffers, Brunner, et al., 2014; Scheffers et al., 2014). A more robust prediction would take into account these differences in warming rate for each microhabitat compared with ambient temperature, which would likely be more favorable for the lizards. Furthermore, we measured burrow *T*_e_ relatively close to the entrances of *D. ingens* burrows, and it is likely that temperatures are lower deeper inside these complex burrow networks. The fact that lizard *T*_b_ was lower than burrow *T*_e_ at night in May (Figure 3) supports this notion. As the climate warms, lizards may be able to move deeper inside these burrows to continue thermoregulating within their *T*_set_.

## 5 CONCLUSION

We found that *G. sila* without access to shrubs are not necessarily in greater danger of overheating or losing hours of activity, as lizards at Shrubless thermoregulated closer to their *T*_set_ than lizards from Shrubbed. While shrubs may play an important role in lizard thermoregulation, lizards at Shrubless spent more time in burrows and thermoregulated more accurately, suggesting that burrows are as important to the thermal ecology of *G. sila* as shrubs, or likely even more important. There also appears to be a trade‐off between more accurate thermoregulation and activity spent aboveground, as implied by the fact that lizards at Shrubless had higher thermoregulatory accuracy and spent more time in burrows. In addition to deploying artificial shade structures (Ghazian et al., 2020), ensuring the continued presence of *D. ingens* may be essential in securing *G. sila* persistence. Burrows excavated by ecosystem engineers such as *D. ingens* are often critical to the survival of other community members (Pike & Mitchell, 2013; Prugh & Brashares, 2012).

Additionally, our data suggest that shrubs could be important in protecting *G. sila* from avian predators such as ravens, further underscoring the conclusion that the ideal habitat for *G. sila* is San Joaquin Desert with *D. ingens* precincts and shrubs. To ensure that our results are relevant to the conservation of *G. sila* across California's San Joaquin Desert, expanding our methods to include additional populations of *G. sila* would provide a management‐applicable understanding of how these lizards interact with their thermal landscape on multiple spatial scales (Steen, 2010). Recognizing the importance of water availability, another environmental factor that is becoming more and more limited in the San Joaquin Desert as droughts become more regular will also help us understand constraints faced by *G. sila* and other desert lizards that are facing similar stressors.

## CONFLICT OF INTEREST

None declared.

## AUTHOR CONTRIBUTIONS

**Nicole Gaudenti:** Conceptualization (supporting); data curation (lead); formal analysis (lead); funding acquisition (supporting); investigation (equal); methodology (equal); project administration (equal); resources (supporting); software (equal); supervision (equal); validation (lead); visualization (lead); writing‐original draft (lead); writing‐review & editing (equal). **Emmeleia Nix:** Data curation (equal); writing‐review & editing (supporting). **Paul Maier:** Data curation (equal); writing‐review & editing (supporting). **Michael F. Westphal:** Conceptualization (equal); funding acquisition (lead); methodology (supporting); project administration (supporting); resources (lead); writing‐review & editing (supporting). **Emily N. Taylor:** Conceptualization (equal); formal analysis (supporting); funding acquisition (equal); methodology (equal); project administration (equal); resources (equal); supervision (lead); writing‐original draft (supporting).

## Data Availability

Dryad doi: https://doi.org/10.5061/dryad.3xsj3txh9.
